# Editorial: Advances in tuberculosis in children: diagnosis, treatment, and management strategies

**DOI:** 10.3389/fped.2026.2009297

**Published:** 2026-10-19

**Authors:** Peize Zhang

**Affiliations:** Department of Pulmonary Medicine & Tuberculosis, Shenzhen Third People’s Hospital, Southern University of Science and Technology, Shenzhen, Guangdong, China

**Keywords:** diagnostic uncertainty, nontuberculous mycobacteria, pediatric tuberculosis, tuberculosis preventive treatment, tuberculous meningitis

Pediatric tuberculosis (TB) remains a major cause of preventable morbidity and mortality. Progress in pediatric TB should be judged by whether children are diagnosed and treated before preventable harm occurs. However, diagnostic uncertainty persists because clinical presentations overlap with common childhood illnesses, microbiological confirmation is difficult, and access to appropriate testing remains uneven (Yang et al.). This Research Topic brings together six contributions spanning diagnostic developments, clinical differentiation, severe disease, host susceptibility, nontuberculous mycobacterial (NTM) disease, and school-based TB control. The Research Topic includes one narrative review, one retrospective cohort study, three case-based contributions, and one outbreak investigation. Together, these articles suggest that improving outcomes requires more than adding another test: it requires connecting diagnostic evidence with timely, individualized care and effective prevention.

## Diagnostic advances in the clinical context

In their narrative review, Yang et al. examined recent developments in molecular diagnostics, host-response biomarkers, and artificial intelligence-assisted imaging. Molecular testing of non-sputum specimens offers opportunities to reduce sampling barriers, whereas newer biomarkers and imaging algorithms still require robust pediatric validation. The distinction between technical promise and clinical usefulness is central. A test that performs well in a selected research population may not retain that performance across different ages, disease presentations, or healthcare settings. Research should therefore address not only diagnostic accuracy but also specimen feasibility, affordability, turnaround time, and whether results lead to earlier treatment. The key question is not whether a test is novel, but whether it changes management and improves outcomes.

Wang et al. addressed the practical difficulty of distinguishing pulmonary TB from pneumonia. In a retrospective cohort of 63 children, their model combining multiple pulmonary nodules and lymphadenopathy showed high discrimination, with a reported area under the receiver operating characteristic curve of 0.99. These findings draw attention to potentially useful imaging clues, but the estimate comes from a small, select population at a specialist hospital and includes only 15 pneumonia controls. Independent prospective validation is needed before the model can guide broader clinical practice. Its present contribution is to inform integrated clinical assessment, not to establish computed tomography as a standalone diagnostic or routine screening strategy.

## Recognizing severe disease and host susceptibility

The consequences of diagnostic uncertainty are particularly apparent in the two TB case reports. Li et al. described infantile endobronchial TB accompanied by *Pneumocystis jirovecii* coinfection and reported an *IKZF1* variant associated with immune dysfunction. Serial bronchoscopic assessments documented necrotizing airway disease and stenosis, and improvement followed combined systemic treatment and bronchoscopic interventions. This case emphasizes the importance of considering airway complications and underlying immune abnormalities in unusually severe infantile disease. However, the favorable outcome cannot isolate the contribution of intraluminal drug administration or establish its ability to prevent permanent stenosis. This aspect requires comparative evidence.

Contreras-Rojas et al. described a toddler with tuberculous meningitis who presented with cerebral infarcts that preceded the appearance of classic basilar enhancement and hydrocephalus. Serial imaging documented substantial progression over 4 days, despite initially normal large-vessel angiography and normal chest radiography. This report illustrates a clinically important limitation of pattern-based recognition: an incomplete early radiological picture does not exclude severe disease. Cerebrospinal fluid findings, epidemiological history, neurological examination, and evolving imaging must be considered together. In this case, management combined antituberculosis treatment, corticosteroids, cerebrospinal fluid diversion, and rehabilitation, illustrating the need for coordinated care beyond antimicrobial prescribing alone.

The imaging cohort and the meningitis case offer a useful contrast: recognizable patterns may help distinguish disease in one selected population, while their early absence can be misleading in a different clinical setting. The value of imaging therefore depends not just on the feature detected, but on disease site, timing, and the population in which it is interpreted (Wang et al., Contreras-Rojas et al.).

Ruan et al. extended the diagnostic discussion to nontuberculous mycobacterial (NTM) disease, an important differential diagnosis rather than a form of TB. Their five-patient series included *Mycobacterium abscessus* and *Mycobacterium paragordonae* infections across different anatomical sites and host conditions, including structural airway disease, cystic fibrosis, and DiGeorge syndrome. Species-level identification helped distinguish clinically similar presentations that required different management. Targeted sequencing was useful in selected cases; however, this series could not establish comparative diagnostic accuracy or treatment superiority. Molecular findings need interpretation alongside clinical, imaging, histopathological, and other microbiological evidence. This contribution also highlights why both pathogen identity and host defenses matter when an apparent respiratory or mycobacterial infection follows an atypical course.

## Linking individual care with prevention

Tang et al. shifted their attention to adolescents in a vocational-school outbreak. Repeated contact screening, classroom and dormitory assessments, and whole-genome sequencing provided complementary information about the cluster. Genomic relatedness supported the epidemiological investigation, but it should not be equated with proof of every transmission link or its direction. The investigation also documented barriers to preventive treatment, including concerns among students, families, and clinicians. Identifying infection is therefore only one step: prevention depends on appropriate assessment, communication, treatment uptake, and follow-up. This study reinforces the need to connect clinical services with schools and public health teams, alongside the implementation of measures to improve ventilation and timely recognition of illness.

## Management implications and unanswered questions

These contributions leave important questions unanswered. None provide a comparative evaluation of pediatric TB regimens or drug exposure. The clinical reports instead show why disease site, organ complications, host factors, and the feasibility of follow-up deserve attention alongside antimicrobial selection. Children with airway disease or neurological complications need coordinated specialist care; adolescents offered preventive treatment need a credible explanation of the benefits and harms. A useful research agenda must address both treatment optimization and the practical conditions required to deliver it.

Future studies should validate diagnostic approaches in diverse pediatric populations, include children with complex comorbidities, and measure their effects on treatment decisions and patient outcomes. Longer-term respiratory and neurological follow-up is needed, alongside an assessment of treatment safety. Implementation studies should examine where children and families are lost between testing, treatment initiation, and completion. The next advance will be meaningful when diagnostic innovation and clinical vigilance are matched by reliable access to care. Earlier recognition, appropriate interpretation of evidence, and sustained linkage to treatment and prevention should define progress in childhood and adolescent TB.

